# A High-Performance Liquid Chromatography with Electrochemical Detection Method Developed for the Sensitive Determination of Ascorbic Acid: Validation, Application, and Comparison with Titration, Spectrophotometric, and High-Performance Liquid Chromatography with Diode-Array Detection Methods

**DOI:** 10.3390/foods12163100

**Published:** 2023-08-18

**Authors:** Fanhua Wu, Fangrui Xu, Wen Liu, Sinan Chen, Haojie Luo, Ni Cheng, Haoan Zhao, Wei Cao

**Affiliations:** 1 College of Food Science and Technology, Northwest University, Xi’an 710069, China; wufanhua_1@163.com (F.W.); 18802945129@163.com (F.X.); liuwen1412@163.com (W.L.); chensinan111@163.com (S.C.); luohj8628@163.com (H.L.); chengni@nwu.edu.cn (N.C.); zhaohaoan@nwu.edu.cn (H.Z.); 2 Bee Product Research Center of Shaanxi Province, Xi’an 710065, China

**Keywords:** ascorbic acid, electrochemical detection, method validation, honey, fruit, biosamples

## Abstract

L-ascorbic acid (vitamin C, VC), an essential nutrient obtained from the diet to maintain various vital signs for the human body, is a crucial indicator of food quality and nutritional value. Herein, high-performance liquid chromatography with electrochemical detection (HPLC-ECD) was developed and validated with the advantages of higher sensitivity, simpler operation processes, and more rapid detection in measuring VC levels in honey samples when compared with the common methods (titration, spectrophotometric, and HPLC-DAD methods). The results of the HPLC-ECD methodological validation showed that the limit of detection (LOD) was 0.0043 µg mL^−1^; the relative standard deviations (RSDs) of the intra- and inter-day values were between 2.51% and 5.15%, and the regression coefficient was >0.999 in the linear range of 0.1 to 20 µg mL^−1^. The validated HPLC-ECD method was also successfully utilized to evaluate the VC levels in different varieties of honey samples with various storage durations as well as in fruit and biological samples. This study provided a perspective for the further accurate determination of VC content in food and biological samples.

## 1. Introduction

Since its discovery by the scientist Albert Szent-Gyrgyi in the 1930s, vitamin C has played an irreplaceable role as an essential nutrient for human beings [1]. From being the antiscorbutic vitamin in the past to being a crucial antioxidant in modern medicine for disease prevention, VC remains one of the most important vitamins, bringing into play critical biological functions within the human body [2,3]. It is of great significance to accurately measure the VC content to evaluate food quality and nutritional value since fruits and vegetables are a high-quality dietary source of VC in food nutrition terms, but VC is easily accumulated or metabolized during the ripening, oxidative browning, and processing of fruits and vegetables [4,5]. However, the lower VC content in some fruits and vegetables (such as bananas and eggplant) is difficult to accurately quantify using common methods.

There are three common methods for the detection of dietary VC at present, namely, 2,6-dichlorophenolindophenol (2,6-DCPIP) titration [6], bathophenanthroline (BP) spectrophotometry [7], and high-performance liquid chromatography with diode-array detection (HPLC-DAD) [8]. Among them, the titration method takes a long time for solution preparation (the 2,6-DCPIP solution needs to be calibrated in a timely manner), and the sample reaction requires more stringent conditions than other methods (the duration of the titration process must be less than 2 min and the matrix pigment may interfere with the color-rendering results). For spectrophotometric determinations, matrix reactions with interfering metal ions transform the BP into colored complexes, negatively affecting the results [9]. The HPLC-DAD method is one of the most common methods of VC determination at present because of the faster analysis time and better selectivity, but it is difficult to accurately measure the VC levels in products with low VC content using the HPLC-DAD method. Thus, for VC, the best-known and prominent nutrient, it is of great significance to develop a more stable, rapid, and high-throughput analysis method.

Our team has been working for many years on the chemistry and nutrition of bee products. One of our technicians, who once measured the VC levels of honey, has been a senior middle school chemistry teacher since graduating. She is well aware of the complexity of the above methods, especially the 2,6-DCPIP titration method involving redox titration (a required chemical analysis method for senior middle schools), but the longer experimental period encourages teachers to impart some relatively advanced technologies to cultivate students’ innovative thinking. Among them, electrochemical methods, especially chromatographic electrochemical methods, are an emerging choice for stable, rapid, sensitive, and high-throughput analysis of VC levels and for students to cultivate their creativity.

HPLC equipped with an electrochemical detector (HPLC-ECD) is a common chromatographic electrochemical method. In the past, we realized the determination of antioxidant compounds in food through the theory of highly sensitive detection of electrochemical active compounds by HPLC-ECD. Taking honey as an example, the polyphenols in honey possess high electrochemical activity (redox characteristics), so the HPLC-ECD method possessing high sensitivity can be used to detect trace compounds [10]. Similarly, as an indispensable nutrient with electrochemical activity, the detection of VC using HPLC-ECD with high sensitivity is of great significance for food quality and nutritional value evaluation. In previous studies, some HPLC-ECD methods were used to measure the VC levels in various samples [11,12]. For example, Gazdik et al. determined the VC content in pharmaceutical preparations, human blood serum, orange (*Citrus aurantium*), and apple (*Malus* sp.) samples using HPLC-ECD after optimizing the experimental conditions and evaluated the recovery of the method [13]. These studies showed that the HPLC-ECD method seems to be suitable for the determination of VC levels.

Therefore, a newly developed HPLC-ECD method for the determination of VC was established in the present study. After method validation, the HPLC-ECD method was compared with the 2,6-DCPIP titration, BP spectrophotometry, and HPLC-DAD methods by using honey as the test samples in VC determination. Furthermore, the application potential of the HPLC-ECD method was evaluated in different varieties of honey and different kinds of fruits as well as biological samples. Our research provided a perspective for the further accurate evaluation of VC levels in food and biological samples by verifying the applicability, reliability, and systematicness of the established HPLC-ECD method for the determination of VC content.

## 2. Materials and Methods

### 2.1. Chemicals and Regents

L-ascorbic acid (VC, purity > 99%), 2,6-dichlorophenolindophenol (2,6-DCPIP, purity > 98%), and 4,7-diphenyl-1,10-phenanthroline (bathophenanthroline, BP, purity > 98%) were all purchased from Sigma-Aldrich Chemical Co. (St. Louis, MO, USA). Methanol (HPLC grade) was obtained from Thermo Fisher Scientific Inc. (Waltham, MA, USA). The other reagents were all obtained from Tianjin Kemiou Chemical Reagent Co. (Tianjin, China). All solutions were prepared using ultrapure water (18.25 MΩ cm) obtained using the ULUPURE water purification system (Chengdu, China).

### 2.2. Honey and Other Samples

All sample information is shown in Supplemental Table S1. The honey samples were directly obtained from beekeepers, and the kiwifruit, durian, and banana samples were all collected from farmers. To validate the method’s sensitivity, biological samples with a low VC content (mice serum and liver tissue) were also utilized, which were obtained from the previous relevant animal experiments of our team [14]. The animal experiments were ethically approved and met the requirements of the Animal Ethics Committee of the Laboratory Animal Center of Northwest University. The honey and fruit samples were stored at room temperature, and all these samples were analyzed within the same period. Moreover, the biological samples (mice serum and liver) were stored at −80 °C until analysis.

### 2.3. Titrimetric Determination of VC

**Figure 1:**
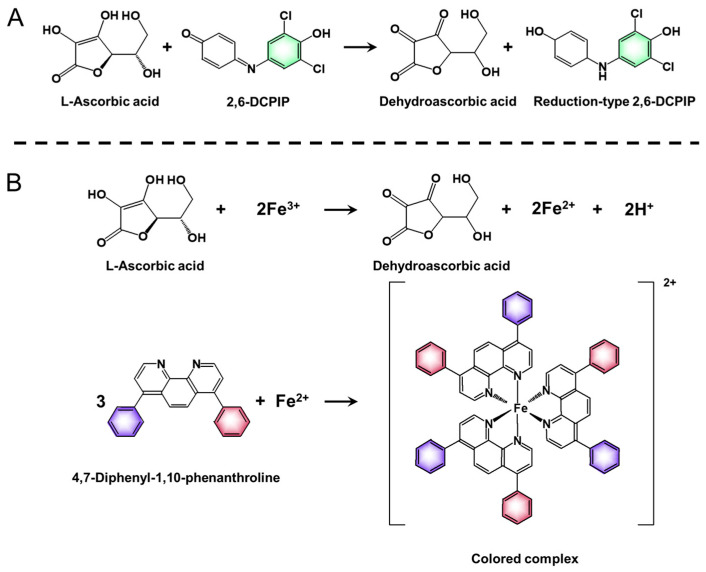
Basic reactions of the ascorbic acid determination by titrimetric and spectrophotometric methods. (**A**) The reaction of ascorbic acid determination by titration based on the conversion of ascorbic acid into its oxidized form (dehydroascorbic acid) and the reduction of 2,6-DCPIP (2,6-dichlorophenolindophenol). (**B**) The reaction of ascorbic acid and Fe^3+^, and the formation of a colored complex between Fe^2+^ and 4,7-diphenyl-1,10-phenanthroline.

The titrimetric determination of VC, based on the reduction of 2,6-DCPIP in a medium containing metaphosphoric acid, was performed according to previous studies [6,15] with minor modifications. The basic reaction is shown in Figure 1A. In this method, 2,6-DCPIP undergoes a reduction reaction with VC, and metaphosphoric acid is used to prevent VC auto-oxidation [16]. The honey samples (1.0 g) were extracted with 1% metaphosphoric acid (10 mL) for 45 min and filtered. The filtrate (1 mL) and blank samples were then titrated with 2,6-DCPIP solution (1 mM). The endpoint of the titration is measured by the solution changing from colorless to pink and holding for more than 15 s. The 2,6-DCPIP solution had to be calibrated with a VC standard solution. The VC content in the measured samples was calculated according to the following formula by comparing with the VC standard:

(1) $$X=\frac{c\times V}{{(V}_{1}-V_{0})}\times \frac{(V_{2}{-V}_{0})\times A}{m}$$

where *X* (mg g^−1^) is the VC content in test samples, and *c* (mg mL^−1^) is the concentration of the VC standard solution. *V* (mL) is the volume of the VC standard solution used to calibrate 2,6-DCPIP. *V*_1_, *V*_2_, and *V*_0_ (mL) are the volumes of 2,6-DCPIP that reacted when titrating the VC standard solution, test samples, and blank solution, respectively. *A* is the dilution multiple of the sample, and *m* (g) is the weight of the test samples.

### 2.4. Spectrophotometric Determination of VC

The spectrophotometric determination of VC was carried out as described by Arakawa et al. with some modifications [7,17]. The method utilized is based on the reduction of Fe (Ⅲ) to Fe (Ⅱ) by VC and the subsequent complex formation with 4,7-diphenyl-1,10-phenanthroline (Figure 1B). The 5% trichloroacetic acid ethanol solution (solution A), 0.4% phosphoric acid ethanol solution (solution B), 0.5% 4,7-diphenyl-1,10-phenanthroline ethanol (solution C), and 0.03% iron trichloride ethanol solution (solution D) were prepared.

The test samples (1 g mL^−1^) were added into a brown test tube, and 1 mL of solution A, 1 mL of absolute ethanol, 0.5 mL of solution B, 1 mL of solution C, and 0.5 mL of solution D were added successively. The test tubes containing the reaction solution were placed in constant-temperature water at 30 °C for 90 min. Subsequently, the absorbance was measured at 534 nm. Aqueous solutions of the VC series standard with concentrations of 5 to 30 mg L^−1^ were used for the calibration, and the absorbance was measured under the same experimental conditions, absorption wavelength, and operation as above. The VC content was measured using a calibration curve with R^2^ = 0.9979 (Table 1). 

### 2.5. HPLC-DAD and HPLC-ECD Methods

The HPLC method described in the previous study [18] with some modifications was used to analyze the VC content. The fruit and biological samples (kiwifruit, durian, banana, and mice liver) had to be homogenized. The 0.1 g test sample was transferred to a 2 mL Eppendorf tube, and 1 mL of 10% metaphosphoric acid solution was added. Then, the mixture was vortexed for 2 min and centrifuged at 12,000 rpm/min at 4 °C for 10 min. The upper supernatant was filtered through a 0.22 μm filter membrane.

HPLC analyses were performed on an Agilent 1290 HPLC system (Agilent, Santa Clara, CA, USA) coupled to a diode-array detector (DAD) and an HP 1049A programmable electrochemical detector (ECD) with an SB-C18 reversed-phase column (Zorbax, 250 mm × 4.6 mm, 5.0 μm). The mobile phase consisted of methanol (solvent B) and mixed aqueous solutions of 50 mM potassium dihydrogen phosphate and 2.5 mM hexadecyl trimethyl ammonium bromide (solvent A). The detecting voltage and the response range for the ECD were held at 900 mV and 500 nA, respectively. The mobile phase was pumped at a flow of 0.7 mL min^−1^ for 7 min with equal gradients of 95% A and 5% B. The column temperature was maintained at 25 °C. The detection wavelength was recorded at 242 nm (for DAD) and the injection volume was 10 μL. VC was identified on the basis of chromatographic retention times and the UV absorption spectrum of the authentic standard. The VC quantitative analysis in each test sample was performed according to the equation of the external calibration curves plotted for the standard solutions with R^2^ > 0.9990 (Table 1).

### 2.6. Method Validation

To investigate and verify the reliability of the methods, the linearity, accuracy, recovery, repeatability, precision, limits of detection (LODs), and limits of quantitation (LOQs) of the methods were evaluated according to the United States Pharmacopoeia Convention [19] and the previous study [20].

The linearity was determined by serial dilutions of a VC standard stock solution of known concentration. The calibration curve was obtained by plotting the absorbance or peak areas of VC versus its concentration. The recovery was evaluated by adding known concentrations of VC standards to honey samples and comparing them with calibration standards of the same concentration. The precision was measured by analyzing three replicates of control samples with known concentrations of VC on three consecutive days. The calculated concentration of VC was compared with the known concentration within intra-day and inter-day periods. The sensitivity was evaluated by determining the limit of detection (LOD) and limit of quantitation (LOQ). The LOD and LOQ are the concentrations at which the signal-to-noise ratio (S/N) is three or ten. The repeatability was tested by measuring six replicates of one test sample. The accuracy was assessed by comparing the measured concentration with an added concentration and calculated using the equation below.

(2) $$Accuracy\left(\%\right)=\frac{Measured\;concentration}{Expected\;concentration}\times 100\%$$

### 2.7. Statistical Analysis

All experiments were analyzed in triplicate, and the results are shown as mean ± standard deviation (SD). Values of *p* < 0.05 were considered to be significant. All calculations and statistical analyses were conducted with SPSS Statistics 17.0 software (SPSS Inc., Chicago, IL, USA) and GraphPad Prism 8.0.2 (GraphPad Software Inc., La Jolla, CA, USA).

## 3. Results and Discussion

### 3.1. Validation of the Analytical Method for VC

The newly developed HPLC-ECD method was validated by assessing the linearity, accuracy, recovery, sensitivity, repeatability, precision, LOD, and LOQ parameters shown in Table 1 and Table 2. Our experimental results indicated that the HPLC-ECD method could efficiently and sensitively analyze VC levels in honey samples. The linearity (calibration curves) was determined from the corresponding relationship between the peak areas and serial concentrations of the VC standard. The linear calibration curve with a wide range of VC concentrations showed favorable linearity (R^2^ > 0.999). The sensitivities were described by the LOD and LOQ. The LOD and LOQ of VC analyzed by the HPLC-ECD method were 0.0043 and 0.0142 μg mL^−1^, respectively. The relative standard deviations (RSDs) obtained from VC concentrations of the test samples for repeatability, intra-day precision, and inter-day precision were 1.36%, 2.51%, and 5.15%, respectively. The recovery values, determined by adding a VC standard with two levels (2 and 4 μg mL^−1^) to the honey samples, were 94.20% and 93.30%, respectively. The distorting matrix effects or losses during sample preparation can be excluded because the detected recoveries were within the range of the accuracy of the method [21]. VC standard solutions with four distinct concentrations (1, 2, 4, and 10 μg mL^−1^) were analyzed to evaluate the accuracy of the methods. The accuracy of the HPLC-ECD method ranged from 96.90% to 102.00%, and the measured value deviated from the true value by <5%, thus indicating true and accurate measurements.

In addition, method validation was carried out on the more extensively used methods (BP and HPLC-DAD) because the BP method for VC determination was also developed for the first time. The sensitivity of the HPLC-DAD and HPLC-ECD methods was compared. The results displayed in Table 1 and Table 2 show that these three methods presented good linearities with high coefficients of determination (R^2^), indicating a good correlation between the experimental measurements and actual values. In the verification study, the results of the examined parameters indicated that the BP and HPLC-DAD methods as well as the HPLC-ECD method were sufficient to analyze the VC content in the test honey samples. However, the BP and HPLC-DAD methods presented unsatisfactory LOD and LOQ values. The LOD values of the BP and HPLC-DAD methods were 2.4167 and 0.9375 μg mL^−1^, respectively, which were much higher than that of the HPLC-ECD method (0.0043 μg mL^−1^). As shown in Table 2, the accuracy was confirmed by spiking the reference standard samples with 1, 2, 4, and 10 μg mL^−1^ of VC standard and performing the recovery method. The accuracy results of the BP and HPLC-DAD methods ranged from 94.45% to 96.73% and from 98.15% to 98.17%, respectively, which were obtained by spiking the reference standard samples with 4 and 10 μg mL^−1^ of VC. However, the accuracy results of the HPLC-ECD method obtained by spiking with 1 and 2 μg mL^−1^ of VC standard, ranged from 97.55% to 102.00%, which also revealed a high sensitivity of the HPLC-ECD method. These results were expected since the HPLC-ECD methods are, generally, more sensitive than the spectrophotometric and HPLC-DAD methods, which is consistent with previous studies [22,23]. Therefore, in comparison, HPLC-ECD is an attractive method with the advantages of high sensitivity, simplicity, and rapid detection.

### 3.2. Comparison of HPLC-ECD and Other Three Methods

**Figure 2:**
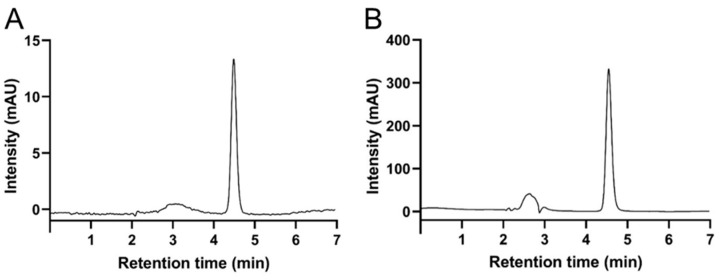
Typical chromatograms of ascorbic acid determined by (**A**) HPLC-DAD and (**B**) HPLC-ECD.

The other three methods, commonly used to measure the VC levels, were compared with the HPLC-ECD method by using honey samples as test sets (Table 3). The results for VC quantification using the 2,6-DCPIP titration, BP spectrophotometry, HPLC-DAD, and HPLC-ECD methods on nine honey samples (H1-H9) from three different botanical origins are shown in Table 3. The results for the VC contents in the same honey sample determined using the HPLC-DAD and HPLC-ECD methods showed similarity, including the standard deviations of the methods. The standard deviations of the 2,6-DCPIP titration and BP spectrophotometry methods were in the range of 0.01 to 0.05 mg g^−1^ and 0.01 to 0.03 mg g^−1^, which were much higher in comparison with those of the HPLC-DAD and HPLC-ECD methods. From the aspect of sensitivity, as shown by the method validation above, the BP and HPLC-DAD methods for VC determination showed higher detection and quantitation limits and lower sensitivities. Under specific experimental conditions, test samples with a VC content higher than 0.1 mg g^−1^ can be quantitated by the 2,6-DCPIP, BP, and HPLC-DAD methods, but the detection could fail or be wildly inaccurate at levels below 0.05 mg g^−1^ (Table 3). As shown in Figure 2, the chromatographic response of HPLC-ECD was tens of times higher than that of HPLC-DAD. From the perspective of accuracy, the deviation in the VC content determined by the HPLC-ECD method is acceptable in the linear range, indicating that the accuracy of the proposed method was at a consistent level with that of the other three methods.

However, both the 2,6-DCPIP and BP methods may easily contribute to false positive reactions. These two methods were not completely specific, making them vulnerable to interference from other factors. For instance, color interferences from samples at the endpoint of the detection analysis and the presence of other oxidizing substances in the samples may affect the accuracy of the results [16]. Especially for the 2,6-DCPIP method, which has been most extensively studied, the titration endpoint of the reaction is determined by the solution changing from colorless to pink, making it difficult to perceive because of the reversible reaction under analytical conditions. And also, when titrating colored or dark-colored honey samples, it is difficult to judge the titration endpoint due to the color interference, resulting in higher standard deviations for the determination of VC content in honey samples. In order to eliminate the color interference of the sample matrix on the experimental results, the sample should be decolorized. As for the BP method, under specific analytical conditions in this study, it requires more than 90 min of reaction time for full-color intensity to ensure the accuracy of experimental results. In addition, this method based on the complexation of reduced Fe (Ⅱ) is nonspecific and, thus, may be interfered with by other reducing agents. For example, in addition to ferrous ions, BP combined with cuprous ions can also form colored complexes to interfere with the determination [16], although such interference did not occur in the present determination.

Generally, HPLC coupled with different detectors has been the most preferred method to measure VC levels [11,24] because of the higher selectivity, specificity, and sensitivity than titration or spectrophotometric methods. HPLC equipped with DAD or ECD has been commonly used for VC determination. No matter what method is used, the most important aspect has always been the accuracy and reliability of the obtained results. However, as shown in Table 3, compared with the HPLC-ECD method under the same experimental conditions, it was hard for the HPLC-DAD method to measure the VC levels of acacia honey samples, which were determined to have a lower VC content ranging from 0.01 to 0.05 mg g^−1^. VC is relatively reactive, and thus, the response signals of VC with electrochemical activity can be significantly improved by ECD (Figure 2), and the chromatographic response of HPLC-ECD was tens of times higher than that of HPLC-DAD. Therefore, compared with the other three methods for VC determination, the HPLC-ECD method can provide the highest sensitivity and specificity as well as reduce the interferences from unrelated substances in the sample [25].

### 3.3. Application of the HPLC-ECD Method for VC Quantitation in Different Samples

VC is one of the crucial components of the non-enzymatic antioxidant defense system in organisms. The accurate determination of changes in the nature or contents of VC will explain the necessity of antioxidant homeostasis [26]. However, based on the results obtained above, it may be difficult for these three common methods involved in this study (2,6-DCPIP titration, BP spectrophotometry, HPLC-DAD) to accurately measure the small variation in VC levels during mild processing and storage conditions as well as the lower VC content in honey samples. In addition to honey samples, we also selected fruits with a higher VC content (kiwifruit) and a lower VC content (durian and banana). The results shown in Table 4 indicate that the proposed HPLC-ECD method was successfully applied for the quantification of VC in honey and fruits as well as in mice serum and liver tissue samples.

#### 3.3.1. Evolution of VC Contents in Honey during Storage

**Figure 3:**
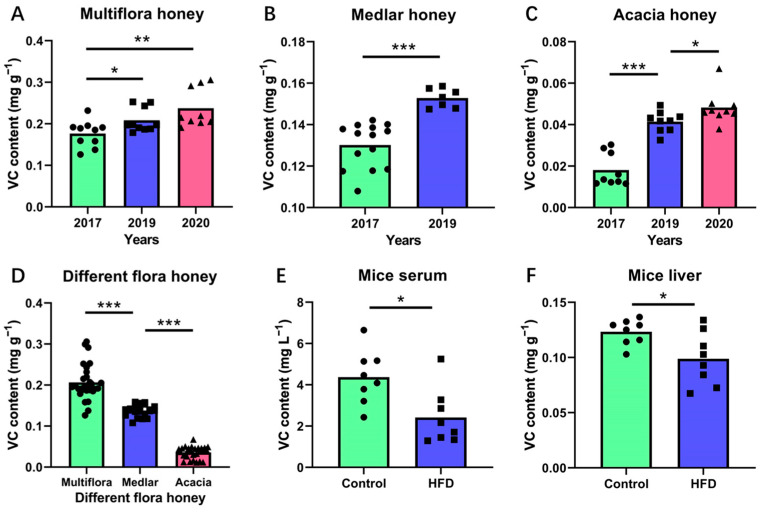
The changes in VC contents in different test samples by using the HPLC-ECD method. Groups with an asterisk (*) indicated significantly different. *, **, and *** indicate *p* < 0.05, *p* < 0.01, and *p* < 0.001, respectively.

The VC content, which is easily affected by processing and storage conditions, has been commonly used as an indicator assessing nutrient loss in foodstuffs [27]. As shown in Figure 3A–C and Table 4, the VC content in honey samples, presented as mg g^−1^, has been found to decrease with the duration of storage. Based on the data of Figure 3A–C and Table 4, higher VC contents were determined in the more recently harvested honey samples. The negative effect of the room temperature storage process on VC content in honey was markedly observed (*p* < 0.05, 0.01, or 0.001). The average values of VC contents in the multifloral, medlar, and acacia honey samples with more recent harvest dates were 0.23, 0.15, and 0.05 mg g^−1^, respectively, while the VC contents in the multifloral, medlar, and acacia honey samples collected in 2017 had mean values of 0.18, 0.13, and 0.02 mg g^−1^, respectively. As seen from the results of VC contents in the same honey sample determined by different methods (Table 3), it is not easy for the 2,6-DCPIP and BP methods to measure the slight variations in VC levels in honey samples during room temperature storage conditions because of the higher standard deviation, and it is also hard for the HPLC-DAD method to determine low VC contents.

The results of the decrease in VC content during storage were consistent with previous studies, and the degradation of VC during food storage and thermal preservation has been proved to follow first-order kinetics [28]. Bosch et al. investigated the changes in VC contents of a fruit-based beikost product after storage in plastic polypropylene/ethylene-vinyl alcohol vacuum packaging at temperatures of 4, 25, 37, and 50 °C for 4, 8, 12, 16, and 32 weeks. The results revealed that the VC degradation followed an Arrhenius first-order kinetic model with an activation energy of 20.11 ± 0.33 kcal mol^−1^ [29]. Wang et al. reported that the VC content in red peppers showed a decreasing trend during 6-month storage [30]. These findings in the current work consistently match the above with the loss of VC content during honey storage.

#### 3.3.2. VC Contents in Different Varieties of Honey

The VC content of honey showed significantly different levels for various botanical origins. As presented in Figure 3D and Table 4, the VC contents in multifloral honey, medlar honey, and acacia honey were in the range of 0.13–0.31, 0.11–0.16, and 0.01–0.07 mg g^−1^, respectively. Alshammari et al. demonstrated that VC content varied in honey with the botanical and geographical origins [31]. Furthermore, León-Ruiz et al. reported that the VC contents in echium honey, lavender honey, and honeydew honey from Spain were 21.80, 21.10, and 7.70 mg kg^−1^, respectively [32]. Among the three varieties of tested honey samples, multifloral honey exhibited the highest average VC content of 0.21 mg g^−1^. This result was in agreement with the previous study. Perna et al. found that multifloral honey presented the highest amount of detected VC content when compared with chestnut honey, eucalyptus honey, citrus honey, and sulla honey [33], which is also a higher level than that of the honey samples from Saudi Arabia [31].

#### 3.3.3. VC Contents in Different Fruit and Biological Samples

In addition to testing honey samples, the HPLC-ECD method was also used to determine the VC levels in fruits, including kiwifruit, durian, and banana, as well as serum and liver obtained from mice. Moreover, because of the limited samples and the trace VC content in biological samples, the HPLC-ECD method was initially used to determine the VC content in blood, urine, and other tissue samples. As depicted in Table 4, the average levels of VC content measured in durian and banana were 0.12 and 0.15 mg g^−1^, which were similar to those in medlar honey. The highest VC level among all test samples was observed in kiwifruit, with a range of 0.94 to 2.05 mg g^−1^. Compared with the VC concentrations in orange samples ranging from 0.30 to 0.56 mg g^−1^ and in apple samples ranging from 0.11 to 0.19 mg g^−1^ as determined by Gazdik et al. [13], the results obtained above indicated that kiwifruit is an excellent source of VC, which was consistent with the previous study [34].

As a non-enzymatic antioxidant, VC can scavenge oxygen free radicals and is also beneficial for the protection of cell function [35]. The results shown in Figure 3E,F indicated that VC contents in the serum and liver tissue of HFD mice were significantly lower than VC content determined in normal groups. Early cohort studies have shown that there is an association between fat accumulation (increased abdominal adiposity) and lower plasma VC concentrations [36]. The increase in plasma fatty acids in the Western diet (high-fat diet) resulted in the activation of the redox cycle of copper–albumin complex and lipid peroxidation [37], which is the potential mechanism to alter the plasma concentrations of VC. Therefore, the VC contents of serum and liver tissue in mice fed with the high-fat diet were significantly lower than those of normal mice in the present study (*p* < 0.05). It has been reported that administration of VC to obese mice alleviated the symptoms of HFD-fed obese mice, such as body weight, visceral adipose tissue mass, and visceral adipocyte size [38].

## 4. Conclusions

The present study developed and validated an HPLC-ECD method with the advantages of high sensitivity, simplicity, rapid detection, and specificity for VC determination in food and biosamples. Based on the experimental data, the 2,6-DCPIP titration, BP spectrophotometry, and HPLC-DAD methods as well as the HPLC-ECD method were all efficient for quantifying VC in honey samples. The results of the HPLC-ECD methodological validation showed that the limit of detection (LOD) was 0.0043 µg mL^−1^; the relative standard deviations (RSDs) of the intra- and inter-day values were between 2.51% and 5.15%, and the regression coefficient was >0.999 in the linear range of 0.1 to 20 µg mL^−1^. Therefore, combined with the methodological validation, the ascorbic acid quantification of nine honey samples from three different botanical origins indicated that the HPLC-ECD method was more sensitive than the other three methods. The results of this study revealed that the developed HPLC-ECD method was successfully applied for the analysis of ascorbic acid in different varieties of honey, fruit, and biological samples, especially applied for the determination of the samples with lower VC content and monitoring the small variation in VC levels during mild processing and storage conditions in honey samples. By verifying the applicability, reliability, and systematicness of the established HPLC-ECD method, a perspective for the further accurate determination of VC content in food and biosamples was provided by this study.

## Figures and Tables

**Table 1:** The linearity and sensitivity of ascorbic acid.

Methodologies	Linearity			LOD ^a^ (μg mL^−1^)	LOQ ^a^ (μg mL^−1^)
	Regression Equation	R^2^	Linear Range (mg mL^−1^)		
BP ^b^	Y = 30.0670x + 2.1160	0.9979	5.0 × 10^−3^–4.0 × 10^−1^	2.4167 (A = 0.01) ^c^	/
HPLC-DAD	Y = 3.0722x + 0.1259	0.9990	5.0 × 10^−3^–2.0 × 10^−2^	0.9375	3.1250
HPLC-ECD	Y = 0.0014x + 0.0423	0.9999	1.0 × 10^−4^–2.0 × 10^−2^	0.0043	0.0142

^a^ Sensitivities are represented by LOD and LOQ. ^b^ BP represents bathophenanthroline spectrophotometry. ^c^ The letter A represents absorbance.

**Table 2:** Accuracy, recovery, repeatability, and precision of ascorbic acid detection methods.

Methodologies	Accuracy (%)				Recovery (%)		Repeatability (n = 6)	Precision	
	Spiked Concentrations (μg mL^−1^)				Spiked Concentrations (μg mL^−1^)		RSD (%)	Intra-Day (n = 6)	Inter-Day (n = 3 Replicates × 3 Days)
	1.00	2.00	4.00	10.00	2.00	4.00		RSD (%)	RSD (%)
BP	/	/	94.45	96.73	/	97.55	5.17	6.53	8.19
HPLC-DAD	/	/	98.17	98.15	94.90	95.63	0.88	3.18	4.49
HPLC-ECD	102.00	97.55	96.90	98.59	94.20	93.30	1.36	2.51	5.15

**Table 3:** The results of ascorbic acid contents in the same honey sample by different methods (mg g^−1^).

Code	Samples	2,6-DCPIP	BP	HPLC-DAD	HPLC-ECD
H1	Multifloral honey	0.20 ± 0.03	0.18 ± 0.01	0.19 ± 0.00	0.18 ± 0.00
H2	Multifloral honey	0.20 ± 0.05	0.19 ± 0.03	0.19 ± 0.00	0.19 ± 0.00
H3	Multifloral honey	0.23 ± 0.05	0.22 ± 0.01	0.22 ± 0.00	0.21 ± 0.00
H4	Medlar honey	0.12 ± 0.05	0.11 ± 0.01	0.12 ± 0.00	0.12 ± 0.00
H5	Medlar honey	0.14 ± 0.01	0.13 ± 0.01	0.13 ± 0.00	0.14 ± 0.00
H6	Medlar honey	0.15 ± 0.05	0.15 ± 0.01	0.15 ± 0.00	0.15 ± 0.00
H7	Acacia honey	ND	ND	ND	0.01 ± 0.00
H8	Acacia honey	ND	ND	ND	0.04 ± 0.00
H9	Acacia honey	ND	ND	ND	0.05 ± 0.00

Data expressed as means ± standard deviation (n = 3). ND, not detected.

**Table 4:** Ascorbic acid contents in honey, kiwifruit, durian, banana, and biological samples by using HPLC-ECD method.

Samples	Geographical Origin	Harvest Date	Mean ± SD	Minimum Value	Maximum Value
Honey (mg g^−1^)					
Multifloral honey (n = 10)	Shaanxi	August 2017	0.18 ± 0.03	0.13	0.23
Multifloral honey (n = 10)	Shaanxi	August 2019	0.21 ± 0.03	0.18	0.25
Multifloral honey (n = 9)	Shaanxi	September 2020	0.23 ± 0.04	0.19	0.31
Medlar honey (n = 10)	Qinghai	November 2017	0.13 ± 0.01	0.11	0.14
Medlar honey (n = 4)	Ningxia	November 2017	0.13 ± 0.01	0.13	0.14
Medlar honey (n = 7)	Qinghai	November 2019	0.15 ± 0.00	0.15	0.16
Acacia honey (n = 9)	Yanan, Shaanxi	April 2017	0.02 ± 0.01	0.01	0.03
Acacia honey (n = 9)	Yanan, Shaanxi	May 2019	0.04 ± 0.00	0.03	0.05
Acacia honey (n = 9)	Yanan, Shaanxi	April 2020	0.05 ± 0.01	0.04	0.07
Fruit (mg g^−1^)					
Kiwifruit (n = 8)	Meixian, Shaanxi	October 2020	1.88 ± 0.13	1.75	2.05
Kiwifruit (n = 8)	Zhouzhi, Shaanxi	October 2020	1.14 ± 0.11	0.94	1.32
Durian (n = 5)	Guangdong	July 2020	0.11 ± 0.01	0.10	0.12
Durian (n = 5)	Hainan	June 2020	0.12 ± 0.01	0.10	0.13
Banana (n = 3)	Guangdong	December 2020	0.14 ± 0.00	0.14	0.14
Banana (n = 6)	Guangxi	December 2020	0.16 ± 0.01	0.15	0.18
Serum (mg L^−1^)					
Control (n = 8)	/	/	4.37 ± 1.22	2.43	6.65
HFD (n = 8)	/	/	2.42 ± 1.27	1.29	5.25
Liver (mg g^−1^)					
Control (n = 8)	/	/	0.12 ± 0.01	0.10	0.14
HFD (n = 8)	/	/	0.10 ± 0.02	0.07	0.13

## Data Availability

All related data that support the findings of this study are contained within this article.

## Supplementary Materials

The following supporting information can be downloaded at https://www.mdpi.com/article/10.3390/foods12163100/s1, Table S1: The information of all test samples.
