# Supplementary material for: A High-Performance Liquid Chromatography with Electrochemical Detection Method Developed for the Sensitive Determination of Ascorbic Acid: Validation, Application, and Comparison with Titration, Spectrophotometric, and High-Performance Liquid Chromatography with Diode-Array Detection Methods
Source: Foods. 2023 Aug 18;12(16):3100. doi: 10.3390/foods12163100 (PMC10453043; doi:10.3390/foods12163100)
Supplement: Supplementary file 1 [file foods-12-03100-s001.zip › foods-2553794-supplementary.pdf]

Table S1. The information of all test samples.

No.	Samples	Species origin	Geographical origin	Harvest date
1	Multifloral honey (n=10)	*Apis cerana*, multifloral	Shaanxi	August 2017
2	Multifloral honey (n=10)	*Apis cerana*, multifloral	Shaanxi	August 2019
3	Multifloral honey (n=9)	*Apis cerana*, multifloral	Shaanxi	September 2020
4	Medlar honey (n=10)	*Apis mellifera*, *Lycium chinense* Mill.	Qinghai	November 2017
5	Medlar honey (n=4)	*Apis mellifera*, *Lycium chinense* Mill.	Ningxia	November 2017
6	Medlar honey (n=7)	*Apis mellifera*, *Lycium chinense* Mill.	Qinghai	November 2019
7	Acacia honey (n=9)	*Apis mellifera*, *Robinia pseudoacacia* L.	Yanan, Shaanxi	April 2017
8	Acacia honey (n=9)	*Apis mellifera*, *Robinia pseudoacacia* L.	Yanan, Shaanxi	May 2019
9	Acacia honey (n=9)	*Apis mellifera*, *Robinia pseudoacacia* L.	Yanan, Shaanxi	April 2020
10	Kiwifruit (n=8)	*Actinidia chinensis* Planch.	Meixian, Shaanxi	October 2020
11	Kiwifruit (n=8)	*Actinidia chinensis* Planch.	Zhouzhi, Shaanxi	October 2020
12	Durian (n=5)	*Durio zibethinus* Murr.	Guangdong	July 2020
13	Durian (n=5)	*Durio zibethinus* Murr.	Hainan	June 2020
14	Banana (n=3)	*Musa nana* Lour.	Guangdong	December 2020
15	Banana (n=6)	*Musa nana* Lour.	Guangxi	December 2020
16	Serum (n=16)	C57BL/6 male mice	Experimental Animal Center of Xi'an Jiaotong University	/
17	Liver tissue (n=16)	C57BL/6 male mice		/
